# The Gut–Extracellular Vesicle–Mitochondria Axis in Reproductive Aging: Antioxidant and Anti-Senescence Mechanisms

**DOI:** 10.3390/antiox15020174

**Published:** 2026-01-28

**Authors:** Efthalia Moustakli, Christina Messini, Anastasios Potiris, Athanasios Zikopoulos, Ioannis Arkoulis, Alexios Kozonis, Theodoros Karampitsakos, Pavlos Machairoudias, Nikolaos Machairiotis, Panagiotis Antsaklis, Periklis Panagopoulos, Sofoklis Stavros, Ekaterini Domali

**Affiliations:** 1 Department of Nursing, School of Health Sciences, University of Ioannina, 4th Kilometer National Highway Str. Ioannina-Athens, 45500 Ioannina, Greece; 2 Department of Obstetrics and Gynaecology, Faculty of Medicine, School of Health Sciences, University of Thessaly, 41500 Larisa, Greece; messini.uth@gmail.com; 3 Third Department of Obstetrics and Gynecology, University General Hospital “ATTIKON”, Medical School, National and Kapodistrian University of Athens, 12462 Athens, Greece; apotiris@med.uoa.gr (A.P.); garkoylis@hotmail.com (I.A.); alkozonis@gmail.com (A.K.); theokarampitsakos@hotmail.com (T.K.); nikolaosmachairiotis@gmail.com (N.M.); perpanag@med.uoa.gr (P.P.); 4 Obstetrics and Gynecology, Royal Cornwall Hospital, Truro TR1 3LJ, UK; thanzik92@gmail.com; 5 Oxford University Hospitals NHS Foundation Trust, Oxford OX3 9DU, UK; paul.machairoudias@ouh.nhs.uk; 6 First Department of Obstetrics and Gynecology, Alexandra Hospital, Medical School, National and Kapodistrian University of Athens, 11528 Athens, Greecekdomali@yahoo.fr (E.D.)

**Keywords:** reproductive aging, extracellular vesicles, gut microbiome, mitochondrial function, oxidative stress, antioxidants, cellular senescence, mitophagy, fertility, reproductive longevity

## Abstract

Cellular senescence, mitochondrial dysfunction, and cumulative oxidative stress (OS) are the main causes of the progressive decreases in oocyte and sperm quality that define reproductive age. There is growing evidence that these processes are controlled by systemic variables, such as metabolites produced from the gut microbiome and extracellular vesicle (EV)-mediated intercellular communication, rather than being exclusively regulated at the tissue level. Antioxidant enzymes, regulatory microRNAs, and bioactive lipids that regulate mitochondrial redox balance, mitophagy, and inflammatory signaling are transported by EVs derived from reproductive organs, stem cells, immune cells, and the gut microbiota. Concurrently, microbiome-derived metabolites such as urolithin A, short-chain fatty acids, and polyphenol derivatives enhance mitochondrial quality control, activate antioxidant pathways, and suppress senescence-associated secretory phenotypes. This narrative review integrates the most recent research on the relationship between redox homeostasis, mitochondrial function, gut microbiota activity, and EV signaling in the context of male and female reproductive aging. We propose an emerging gut–EV–mitochondria axis as a unified framework through which systemic metabolic and antioxidant signals affect gamete competence, reproductive tissue function, and fertility longevity. Finally, we discuss therapeutic implications, including microbiome modulation, EV-based interventions, and senotherapeutic strategies, highlighting key knowledge gaps and future research directions necessary for clinical translation.

## 1. Introduction

Reproductive aging is one of the earliest and most pronounced manifestations of human biological aging. Women gradually lose ovarian reserve and oocyte quality long before somatic decline is clinically apparent, which results in decreased fertility and impaired embryonic competence [1,2,3]. In men, aging is associated with reduced sperm motility, disrupted chromatin integrity, and poor mitochondrial function, reflecting cumulative molecular damage. The progressive accumulation of oxidative stress (OS) and mitochondrial dysfunction is a common biological characteristic underlying reproductive aging, despite the fact that the timing and clinical manifestation vary between sexes [4,5,6,7].

Reactive oxygen species (ROS) are unavoidable byproducts of cellular metabolism and play essential roles in normal reproductive processes, including steroidogenesis, gamete maturation, and embryo development [8,9]. However, when ROS production exceeds antioxidant capacity, oxidative damage accumulates, affecting DNA, lipids, and proteins. Due to their heavy reliance on mitochondrial oxidative phosphorylation for energy production, oocytes, spermatogenic cells, and early embryos are especially susceptible to redox imbalance [10,11]. Gamete competency and reproductive potential are directly impacted by age-related deficits in mitochondrial dynamics, such as decreased mitophagy, altered biogenesis, and damage to mitochondrial DNA (mtDNA) [4,7,12,13].

Beyond cell-intrinsic mechanisms, it is increasingly recognized that reproductive aging is influenced by systemic metabolic and inflammatory signals. The gut microbiome has emerged as a critical regulator of host redox homeostasis and mitochondrial function through the production of bioactive metabolites, such as short-chain fatty acids (SCFAs), urolithins, indole derivatives, and polyphenol metabolites [14,15]. These compounds can cross the intestinal barrier and modulate antioxidant defenses, inflammatory pathways, mitophagy, and mitochondrial biogenesis in distant organs. Dysbiosis, which becomes more prevalent with age, metabolic disease, and environmental stress, is associated with elevated systemic OS, chronic inflammation, and accelerated biological aging. However, little is known about how gut-derived signals affect reproductive organs [14,16,17,18,19,20].

Extracellular vesicles (EVs) are a new layer of mitochondrial and redox control. Released by nearly all cell types, EVs carry a diverse cargo of antioxidant enzymes, bioactive lipids, proteins, and regulatory RNAs capable of modifying mitochondrial function, inflammatory signaling, and cellular stress responses in recipient cells [21,22]. Within the reproductive system, EVs derived from follicular fluid, oviductal and uterine secretions, seminal plasma, and epididymal fluid play essential roles in gamete maturation, fertilization, embryo development, and implantation. Increased OS and poor reproductive results have been associated with age-related changes in EV abundance and cargo composition [23].

Despite significant progress in comprehending the distinct functions of gut microbiome-derived metabolites, mitochondrial dysfunction, and EV-mediated communication in aging and reproduction, these domains have essentially developed concurrently. There is currently no comprehensive paradigm that explains how EV-mediated transport and gut-derived metabolic signals interact to regulate mitochondrial redox and cause cellular senescence in reproductive organs [22,24,25].

This narrative review summarizes the most recent research on the relationship between OS, mitochondrial function, gut microbiome activity, and EV signaling in the context of male and female reproductive aging. We propose an emerging gut–EV–mitochondria axis as a conceptual model through which systemic antioxidant and metabolic signals influence gamete quality, reproductive tissue integrity, and fertility longevity. We conclude by discussing therapeutic implications and highlighting key knowledge gaps that need to be addressed to translate this concept into strategies for maintaining reproductive healthspan.

## 2. Methods

This narrative review aimed to synthesize current evidence on the relationships between gut microbiota–derived metabolites, EV–mediated signaling, mitochondrial function, OS, and cellular senescence in the context of male and female reproductive aging.

A comprehensive literature search was conducted in PubMed, Scopus, and Web of Science databases for articles published until December 2025. The search strategy combined keywords and Medical Subject Headings (MeSH), including reproductive aging, extracellular vesicles, mitochondria, oxidative stress, gut microbiota, senescence, fertility, oocytes, sperm, and assisted reproduction, using Boolean operators as appropriate.

Primary experimental studies (in vitro, in vivo, animal, and clinical investigations) and clinically relevant translational research published in English were prioritized to support mechanistic and functional conclusions. Review articles were included selectively to provide conceptual background and to contextualize emerging themes.

Studies were included if they addressed mitochondrial function, redox regulation, EV signaling, or gut microbiome activity in relation to reproductive biology or aging. Articles not relevant to reproductive systems or lacking mechanistic or translational relevance were excluded. Additional references were identified through manual screening of the reference lists of relevant publications. Owing to the narrative design of this review, no formal systematic review methodology, quality scoring, or meta-analytic approach was applied.

## 3. The Gut–Mitochondria Axis in Reproductive Aging

Mitochondrial function is a key factor of reproductive health, influencing oocyte maturation, sperm motility, steroidogenesis, and early embryonic development [5]. Reproductive tissues are especially vulnerable to OS and mitochondrial malfunction because gametes and preimplantation embryos primarily rely on oxidative phosphorylation to meet their energy needs. Declining reproductive capability is consequently directly linked to age-related deficits in mitochondrial dynamics, such as decreased mitophagy, altered biogenesis, and accumulation of mitochondrial DNA damage [4,7,12,13].

Mitochondrial dysfunction in reproductive aging has traditionally been considered a cell-autonomous process caused by metabolic fatigue and inherent oxidative damage [26]. However, increasing evidence suggests that mitochondrial homeostasis is also shaped by systemic signals originating outside reproductive tissues. Through metabolic, immunological, and inflammatory pathways, the gut microbiome has emerged as a crucial systemic regulator of host redox balance and mitochondrial homeostasis [17,24,25,27].

### 3.1. The Gut Microbiome as a Regulator of Systemic OS

By producing bioactive compounds that modulate mitochondrial function, inflammatory tone, and antioxidant defenses, the gut microbiota significantly contributes to systemic redox homeostasis. Microbial products promote redox equilibrium in metabolically balanced states by boosting glutathione synthesis, reducing excessive ROS generation, and activating antioxidant transcriptional pathways [17,24,28]. In contrast, endotoxemia, increased intestinal permeability, and age-related imbalances are associated with chronic low-grade inflammation and increased systemic OS, which are characteristics of biological aging [18].

Given the reproductive organs’ high susceptibility to oxidative injury, such systemic redox disruptions are especially relevant. Increased levels of ROS and circulating inflammatory mediators can impair mitochondrial integrity and cellular function in germ cells, thereby interfering with spermatogenesis and follicular development [18]. Although direct causal pathways remain under investigation, accumulating evidence supports a connection between gut microbial imbalance, OS, and adverse reproductive outcomes [16].

### 3.2. Microbiome-Derived Metabolites Modulating Mitochondrial Dynamics

Signals from the gut microbiota influence key components of mitochondrial quality control, including biogenesis, membrane integrity, and mitophagy, as well as overall redox regulation. These processes are essential for maintaining mitochondrial efficiency and limiting ROS production, particularly in energy-demanding reproductive cells [29].

Cellular energy metabolism and oxidative resilience are influenced by microbial metabolites derived from dietary components that either directly or indirectly alter mitochondrial signaling pathways. The gut microbiota may support gamete maturation and prevent age-related mitochondrial decrease in reproductive organs through several systemic mechanisms [30,31]. Section 3 provides a detailed discussion of the distinct roles of major metabolite types in regulating reproductive mitochondrial function.

### 3.3. Impact on Female Reproductive Aging: Ovarian Reserve and Oocyte Competence

Progressive loss of oocyte quality and reduced ovarian reserve are hallmarks of ovarian aging and are closely associated with OS and mitochondrial dysfunction [32]. Oocytes are particularly susceptible to redox imbalance due to their high mitochondrial content. Preclinical studies indicate that changes in gut microbiota composition can influence granulosa cell survival, follicular growth, and mitochondrial function, including membrane integrity, through systemic metabolic and inflammatory signaling [33]. Collectively, these results provide credence to the idea that interactions between the gut and mitochondria may play a role in ovarian aging, even if there is still a dearth of human evidence.

### 3.4. Impact on Male Reproductive Aging: Mitochondrial Dysfunction in Sperm

Spermatozoa rely on mitochondrial activity to sustain motility and maintain DNA integrity. Reduced sperm count, increased DNA fragmentation, decreased motility, and compromised chromatin packaging are consequences of dysbiosis-related systemic inflammation and OS. Furthermore, redox imbalance disrupts mitochondrial signaling pathways in Sertoli and Leydig cells that promote steroidogenesis and spermatogenesis. These findings suggest that changes in systemic oxidative state caused by the gut microbiota may have an indirect impact on male reproductive aging [34,35,36].

### 3.5. Integrating the Gut–Metabolite–Mitochondria Model in Reproductive Aging

Collectively, current evidence supports the idea that the gut microbiome functions as a distant regulator of mitochondrial function in reproductive tissues. The gut–mitochondria axis influences reproductive aging by regulating mitophagy, mitochondrial biogenesis, antioxidant gene expression, and ROS levels [24]. This gut–mitochondria axis provides a valuable framework for comprehending how metabolic health and aging interact with fertility and reproductive longevity, although many underlying molecular mechanisms are still unclear [37].

## 4. Gut Metabolites and Reproductive Mitochondria

The gut microbiota modulates mitochondrial activity and systemic redox equilibrium through important mediators known as microbiome-derived metabolites. Many of these compounds have cytoprotective, anti-inflammatory, and antioxidant effects that directly or indirectly regulate mitochondrial dynamics, including membrane integrity, biogenesis, and mitophagy. Alterations in metabolite availability may therefore have a disproportionate impact on reproductive aging, consistent with emerging systems-level models linking metabolism, mitochondrial regulation, and aging [17,38].

### 4.1. Urolithins and Mitophagy

Urolithin A (UA) is one of the most thoroughly studied microbiome-derived regulators of mitochondrial quality control and is produced by specific gut bacteria from dietary ellagitannins. UA mainly triggers mitophagy through the activation of the PINK1–Parkin pathway, encouraging the selective removal of dysfunctional mitochondria and limiting excessive ROS production [39]. In several preclinical models, UA has been shown to improve mitochondrial efficiency, preserve ATP production, and reduce markers of cellular senescence, such as p16INK4a expression and elements of the senescence-associated secretory phenotype (SASP) [40].

Experimental research indicates that UA may promote ovarian function by maintaining oocyte mitochondrial integrity and enhancing meiotic fidelity, despite the lack of direct clinical proof in human reproduction [41]. In line with its function in enhancing mitochondrial quality control, UA has been linked to increased sperm mitochondrial activity and motility in male models. These findings underscore the need for focused reproductive research while positioning UA as a potential mediator linking gut microbiota activity, mitophagy, and reproductive lifespan [13].

### 4.2. SCFAs and Mitochondrial Biogenesis

Microbial fermentation of dietary fibers produces short-chain fatty acids (SCFAs), such as acetate, propionate, and butyrate, which have a wide range of impacts on host metabolism and redox equilibrium [42]. Among these, butyrate is important for controlling antioxidant defenses and mitochondrial biogenesis. Acting as a histone deacetylase inhibitor, butyrate enhances the transcription of mitochondrial regulators such as PGC-1α, NRF1, and TFAM, thereby supporting mitochondrial biogenesis and function [43].

Additionally, SCFAs alleviate OS in reproductive cells by promoting intracellular glutathione synthesis and activating redox-sensitive pathways, including Nrf2 signaling [28]. Experimental evidence indicates that SCFAs enhance sperm mitochondrial membrane potential, granulosa cell viability, and follicular development. Therefore, decreased mitochondrial activity and reproductive aging may be linked to age-related decreases in SCFA-producing bacterial communities [44].

### 4.3. Tryptophan-Derived Metabolites and Redox Signaling

By activating the aryl hydrocarbon receptor (AhR), the microbial breakdown of dietary tryptophan produces a variety of indole derivatives that affect OS and mitochondrial function. In both ovarian and testicular cells, properly controlled AhR signaling improves mitochondrial stability, inhibits inflammatory pathways, and increases the expression of antioxidant genes [45,46].

Disruptions in tryptophan metabolism, commonly observed during gut dysbiosis, have been associated with reduced antioxidant capacity in reproductive tissues, increased granulosa cell apoptosis, and impaired steroidogenesis. In the male reproductive system, decreased synthesis of microbial indole derivatives has been linked to higher DNA fragmentation and increased OS in sperm. Collectively, these results imply that microbiota-driven tryptophan metabolites play a significant role in regulating reproductive redox homeostasis [47,48].

### 4.4. Polyphenol-Derived Metabolites and Mitochondrial Protection

Dietary polyphenols are extensively metabolized by the gut microbiota into smaller, more bioavailable compounds with enhanced biological activity. These polyphenol-derived metabolites exhibit antioxidant properties by stabilizing mitochondrial membranes, reducing lipid peroxidation, and activating sirtuin-dependent pathways, particularly SIRT1 and SIRT3 [49,50].

Polyphenol-derived metabolites have been shown in experimental models to enhance oocyte maturation, preserve sperm chromatin integrity, and maintain redox balance in granulosa cells. These metabolites suggest a significant connection between diet, gut microbiome function, and mitochondrial health in reproductive aging, although their translation to human reproductive outcomes remains under active investigation [51].

### 4.5. Combined Antioxidant Effects on Reproduction

Collectively, microbiome-derived metabolites contribute to an antioxidant environment that supports mitochondrial resilience within reproductive tissues [24,28,39,52]. Despite acting through distinct signaling pathways, urolithins, SCFAs, indole derivatives, and polyphenol metabolites converge on common targets, including mitophagy activation, mitochondrial biogenesis, ROS reduction, and redox-sensitive transcriptional regulation [39,43,49,52]. Although the extent of clinical validation varies among metabolite classes, the mechanisms described above are supported by a substantial body of in vitro, in vivo, and animal experimental evidence linking microbiome-derived metabolites to mitochondrial regulation and redox control in reproductive systems.

As summarized in Table 1, these metabolite-mediated mechanisms translate into improved oocyte quality, enhanced sperm motility and genomic integrity, and a more favorable environment for early embryonic development. Therefore, this beneficial metabolic signaling may be disrupted by age-related changes in microbiome composition, hastening mitochondrial dysfunction and reproductive decline.

## 5. EVs in Redox and Mitochondrial Regulation

EVs are membrane-bound nanoparticles released by nearly all cell types and represent a fundamental mechanism of intercellular communication. EVs, including exosomes and microvesicles, transport a diverse cargo of proteins, lipids, metabolites, and nucleic acids that can modulate OS responses, mitochondrial function, and cellular aging in recipient cells. Increasing evidence indicates that EV-mediated signaling plays a central role in coordinating redox homeostasis and mitochondrial quality control across tissues, including the reproductive system [22,56].

### 5.1. EV Cargo and Mitochondrial Redox Control

A defining feature of EVs is their ability to deliver antioxidant and mitochondrial regulatory components directly to target cells. EV cargo frequently includes antioxidant enzymes such as superoxide dismutase, catalase, glutathione peroxidase, and peroxiredoxins, which can reduce intracellular ROS levels and protect mitochondrial membranes from oxidative damage. In parallel, EVs transport bioactive lipids and regulatory microRNAs that influence mitochondrial biogenesis, oxidative phosphorylation, and mitophagy [57,58].

MicroRNAs enriched in EVs can target key mitochondrial regulators, including components of the PGC-1α signaling axis, sirtuin pathways, and mitophagy-related proteins. Through these mechanisms, EVs can shift recipient cells toward a more antioxidant and metabolically resilient state [59].

### 5.2. EVs as Modulators of OS Responses

EV release and cargo composition are dynamically regulated by the metabolic and redox status of the parent cell. Under conditions of controlled OS, EVs may function as adaptive signaling units, distributing antioxidant capacity and stress-response signals to neighboring or distant cells. Conversely, excessive or chronic OS can alter EV cargo toward pro-inflammatory and pro-oxidant profiles, contributing to redox imbalance in recipient tissues [60].

In vivo, these redox-driven changes in EV cargo often occur in parallel with cytokine and hormone signaling. EV-associated inflammatory mediators, such as cytokines and chemokines, as well as hormone-responsive regulatory molecules, can modulate mitochondrial activity and oxidative stress responses in recipient cells, thereby linking immune and endocrine cues to cellular redox homeostasis [61,62,63].

This dual role underscores the importance of EV quality rather than EV abundance alone. Age-related changes in EV cargo composition, including reduced antioxidant enzyme content and increased inflammatory mediators, have been associated with impaired mitochondrial function and heightened OS in multiple tissues. These shifts may contribute to the propagation of redox imbalance during aging, including within reproductive organs [64,65].

### 5.3. EVs and Mitochondrial Quality Control

EVs impact mitochondrial quality control mechanisms, which are essential for maintaining cellular function as we age, in addition to scavenging ROS. Fission, fusion, biogenesis, and mitophagy are among the processes in mitochondrial dynamics that are regulated by EV-associated microRNAs and proteins. EVs reduce ROS leakage and maintain bioenergetic efficiency by facilitating the elimination of damaged mitochondria and promoting the regeneration of healthy mitochondrial populations [66,67].

EVs produced from metabolically sound or stem cell-rich sources have been demonstrated in experimental models to improve ATP synthesis, lessen oxidative damage in recipient cells, and restore mitochondrial membrane potential [68]. Collectively, these findings support a potential role for EV-mediated signaling in modulating age-related mitochondrial deterioration in reproductive tissues.

### 5.4. EVs as Regulators of Cellular Senescence

Cellular senescence is primarily caused by OS and mitochondrial malfunction, both of which are influenced by EVs. These can modulate cellular senescence by delivering mitochondrial regulators, antioxidant enzymes, and microRNAs that suppress DNA damage responses and limit activation of senescence-associated pathways, including p16^INK4a and p21 signaling. Furthermore, EVs may influence inflammatory signaling in tissues by modifying the SASP [69,70,71].

Notably, in aging models, EVs derived from young or regenerative cell populations have been shown to reduce senescence markers and improve mitochondrial function. However, pro-inflammatory and pro-oxidant EVs are released by senescent cells, which may increase OS and spread senescence. Thus, tissue aging trajectories, including reproductive aging, may be shaped by the equilibrium between these opposing EV populations [57,65,72].

### 5.5. Relevance to Reproductive Aging

Within the reproductive system, EV-mediated regulation of redox balance and mitochondrial function is particularly consequential. Because they are extremely vulnerable to oxidative damage and mitochondrial malfunction, oocytes, spermatogenic cells, and early embryos rely on precise intercellular communication pathways. EVs provide a mechanism for distributing mitochondrial regulatory signals and antioxidant capacity across reproductive tissues and systemic compartments [73,74].

Although many aspects of EV-mediated redox regulation in reproduction remain under investigation, current evidence supports a role for EVs as important modulators of mitochondrial resilience and OS during reproductive aging. These general mechanisms provide the foundation for understanding the tissue-specific roles of EVs in the ovary, testes, and reproductive tract, which are discussed in the following section [75]. While many mechanistic insights derive from non-reproductive models, accumulating experimental evidence indicates that EV-mediated redox and mitochondrial regulation are highly relevant to reproductive tissues, particularly under conditions of aging and OS.

## 6. EVs in the Reproductive Tract

The reproductive tract releases EVs that have specific functions in promoting early embryonic development, aiding fertilization, and preserving gamete quality. Reproductive tract-derived EVs function in highly controlled local microenvironments with high metabolic demand and OS susceptibility, in contrast to circulating EVs that mediate systemic signaling. Therefore, the antioxidant and mitochondrial regulatory functions of these EVs are critical for maintaining reproductive competence, particularly during aging [73,76].

### 6.1. Follicular Fluid EVs and Oocyte Maturation

Granulosa cells, cumulus cells, and the oocyte itself discharge a large number of EVs into the ovarian follicular fluid. These EVs carry mitochondrial regulators, antioxidant enzymes, and microRNAs that regulate oocyte maturation and guard against oxidative damage during folliculogenesis. During meiotic progression, EV-mediated transport of antioxidant components supports ATP synthesis, limits ROS buildup, and preserves mitochondrial membrane potential [58,77].

Follicular development and oocyte competence have been linked to age-related changes in follicular fluid EV cargo, such as decreased antioxidant capacity and enhanced pro-inflammatory signaling. Restoring antioxidant EV signaling may enhance mitochondrial function and oocyte quality in models of ovarian aging, according to experimental research employing EVs produced from metabolically sound or stem cell-enriched sources [78,79].

### 6.2. Oviductal and Endometrial EVs in Early Development

EVs generated by the uterine and oviductal epithelium help provide a favorable environment for gametes and developing embryos after ovulation and fertilization. During the early phases of embryonic cleavage, when intrinsic antioxidant capacity is limited, oviductal EVs transport proteins, lipids, and regulatory RNAs that improve antioxidant defenses and stabilize mitochondrial activity [80,81].

Endometrial EVs also influence implantation and early placental development by modulating redox signaling at the maternal–embryonic interface. Increased OS and decreased reproductive success are linked to disruptions in the composition of oviductal or uterine EVs, such as those seen in endometriosis, polycystic ovarian syndrome, obesity, and advanced maternal age [82,83].

### 6.3. Seminal Plasma and Epididymal EVs in Sperm Protection

EVs found in the seminal plasma and epididymis are essential for shielding spermatozoa from oxidative damage in the male reproductive system. Mature sperm get antioxidant enzymes, membrane-stabilizing proteins, and regulatory RNAs from epididymosomes, which sustain chromatin integrity, motility, and mitochondrial membrane potential [84,85,86].

When sperm are exposed to OS during ejaculation and transit via the female reproductive tract, seminal plasma EVs offer further protection. Increased sperm ROS levels, DNA breakage, and decreased fertilizing ability have all been connected to lower antioxidant EV content, especially in aging, metabolic disorders, and inflammatory conditions [87].

### 6.4. Embryo-Derived EVs and Maternal Communication

Preimplantation embryos release EVs that participate in bidirectional communication with maternal tissues and reflect embryonic metabolic and redox status. These embryo-derived EVs contain mitochondrial components, metabolic enzymes, and stress-responsive microRNAs that may influence immunological tolerance and endometrial receptivity. Emerging evidence suggests that embryo-derived EVs contribute to shaping the oxidative environment required for successful implantation and early embryonic development, although this field remains at an early stage of investigation [88,89].

### 6.5. Age-Related Changes in Reproductive Tract EVs

The composition and functional ability of EVs in the reproductive tract change with age, with an increase in inflammatory content and a decrease in antioxidant signaling. These alterations may increase OS, compromise mitochondrial integrity in gametes and embryos, and ultimately reduce fertility. Understanding how aging reshapes EV-mediated redox signaling within reproductive tissues may inform the development of EV-based or antioxidant-targeted therapeutic strategies [57,65,71].

As summarized in Table 2, reproductive tract–derived EVs serve as local mediators of mitochondrial protection, redox balance, and intercellular communication essential for maintaining fertility across the reproductive lifespan.

## 7. Senescence in Reproductive Aging

A key indicator of aging is cellular senescence, which is marked by irreversible cell-cycle arrest, metabolic reprogramming, and the formation of an SASP. Cellular senescence plays beneficial roles in tissue remodeling and tumor suppression; however, its accumulation in reproductive tissues impairs physiological function and accelerates reproductive decline [12,57].

In ovarian, testicular, and reproductive tract cells, persistent oxidative damage triggers DNA damage responses and redox-sensitive signaling pathways that encourage senescence. Senescent cells further intensify tissue dysfunction via SASP-mediated inflammation, resulting in a self-amplifying cycle that compromises reproductive tissue integrity [92].

### 7.1. Mitochondrial Dysfunction in Ovarian Senescence

The high mitochondrial content and metabolic demands of oocytes and the surrounding somatic cells make the ovary especially vulnerable to senescence-driven aging. Granulosa and cumulus cells produce more ROS as a result of age-related reductions in mitophagy, mitochondrial biogenesis, and mtDNA integrity. These alterations stimulate the release of SASP factors that hinder follicular growth and oocyte maturation and activate senescence markers, including p16^INK4a and p21 [93].

By elevating OS and inflammatory signaling, senescent granulosa cells modify the follicular milieu, jeopardizing oocyte ATP availability, mitochondrial membrane potential, and meiotic spindle integrity. Consequently, decreased ovarian reserve and decreased oocyte competence are intimately associated with the buildup of senescent cells inside the ovarian niche [12].

### 7.2. Testicular Senescence and Sperm Decline

Leydig cells, Sertoli cells, and germ cells in males are impacted by senescence-related mitochondrial malfunction, which contributes to age-related decreases in spermatogenesis and testosterone production. Aging testicular cells’ mitochondria show decreased membrane potential, increased ROS leakage, and decreased oxidative phosphorylation efficiency. These changes encourage SASP-driven inflammation in the testicular microenvironment and trigger senescence pathways [94,95].

Reduced motility, increased DNA fragmentation, and compromised chromatin packaging in spermatozoa are directly linked to mitochondrial dysfunction and OS. Metabolic disorders, inflammation, and exposure to toxins are examples of environmental stressors that worsen mitochondrial ROS production and hasten the reduction in reproduction associated with senescence [6,96].

### 7.3. SASP-Mediated Amplification of OS

Crucially, SASP components have the ability to function in a paracrine fashion, extending oxidative damage beyond the initial senescent population and causing secondary senescence in nearby cells. Age-related reproductive tissue failure is exacerbated by this spread of redox imbalance [66,71].

### 7.4. Modulation of Senescence by Gut-Derived Signals and EVs

Recent evidence indicates that systemic metabolic and redox signals, in addition to local mitochondrial dysfunction, influence senescence in reproductive organs. Microbiome-derived compounds with antioxidant and mitophagy-enhancing properties may attenuate senescence by lowering mitochondrial ROS and suppressing inflammatory signaling, whereas dysbiosis-associated metabolic alterations may accelerate senescence [97].

EVs further modulate senescence by transporting antioxidant enzymes, mitochondrial regulators, and regulatory microRNAs that influence DNA damage responses and SASP expression. While EVs released by senescent cells may propagate pro-senescent signals, EVs derived from youthful or metabolically healthy cells have demonstrated the capacity to reduce senescence markers and restore mitochondrial function in reproductive tissues [72,98].

### 7.5. Targeting Senescence in Reproductive Aging

Strategies aimed at reducing the burden of senescent cells or modulating senescence-associated secretory phenotype (SASP) activity are gaining increasing attention, given the central role of senescence in reproductive decline. Senolytic and senomorphic substances, including naturally occurring antioxidants like quercetin and fisetin, have demonstrated potential in experimental models by reducing OS and improving reproductive tissue function. Additional approaches that enhance mitochondrial quality control, including activation of mitophagy and regulation of antioxidant pathways, may further limit senescent cell accumulation [12,93,99].

As summarized in Table 3, senescence-driven mitochondrial dysfunction and OS contribute directly to impaired gametogenesis, hormonal imbalance, and reduced fertility. Understanding how senescence intersects with gut-derived metabolic signals and EV-mediated communication is therefore critical for developing interventions aimed at preserving reproductive healthspan.

## 8. Gut–EV Communication in Reproductive Aging

There is growing evidence that the gut microbiota affects reproductive aging through endocrine, inflammatory, and metabolic pathways. However, little is known about how gut-derived signals are transmitted to distant reproductive organs [20]. Emerging studies suggest that EVs may contribute to this long-distance communication by transporting regulatory, mitochondrial, and antioxidant signals from the gut and systemic circulation to peripheral tissues, including reproductive organs [91,108].

To preserve a clear mechanistic thread, we conceptualize the proposed axis as a stepwise sequence: (i) gut microbiota–derived metabolites and microbial vesicles modulate systemic redox balance and host EV biogenesis and cargo composition; (ii) these EV populations enter the circulation and reach reproductive tissues; and (iii) EV cargo influences mitochondrial quality control, redox signaling, and stress responses in germ cells and supporting somatic cells, thereby shaping cellular senescence and reproductive aging. While direct experimental validation of each step in reproductive organs is currently limited, mechanistic evidence from multiple biological systems supports the individual components of this pathway.

### 8.1. Gut-Derived Metabolites as Modulators of EV Cargo

EV biogenesis and cargo composition are also influenced by microbiome-derived metabolites, including urolithin A, short-chain fatty acids, and chemicals produced from polyphenols, which are known to control mitochondrial activity and redox equilibrium. EV release and molecular loading are mostly determined by cellular redox status and mitochondrial activity [109,110]. Cells often create EVs rich in anti-inflammatory lipids, regulatory microRNAs, and antioxidant enzymes when metabolic homeostasis is maintained. Conversely, OS and dysbiosis-associated inflammation can shift EV cargo toward pro-inflammatory and pro-senescent profiles [111,112].

Through these mechanisms, gut microbiome activity may indirectly shape the “quality” of EV-mediated signaling received by reproductive cells by altering systemic redox tone. This process provides a plausible link between microbial metabolism and EV-mediated regulation of mitochondrial function, although direct experimental evidence in reproductive contexts remains limited and warrants further investigation [63].

### 8.2. Microbial-Derived EVs as Systemic Redox Signals

In addition to modulating host-derived EV profiles, the gut microbiota itself produces EVs capable of crossing intestinal epithelial barriers and entering the systemic circulation. These microbiota-derived EVs contain lipids, metabolites, and short RNAs that regulate host immune responses, redox signaling, and mitochondrial activity. Notably, microbial EVs can reach distant organs and alter host cellular pathways in non-reproductive systems [113,114,115].

Although their direct effects on reproductive tissues have not yet been fully characterized, the ability of microbiota-derived EVs to influence systemic inflammatory and oxidative states suggests that they may indirectly shape the reproductive microenvironment, particularly under conditions of metabolic stress or aging [108,114].

### 8.3. EV-Mediated Transfer of Antioxidant and Mitochondria Regulators

EVs originating from the gut, immune system, or circulation can transport antioxidant enzymes, mitochondrial regulators, and microRNAs to reproductive cells, including oocytes, granulosa cells, spermatogenic cells, and early embryos. These cargos target key pathways involved in mitochondrial biogenesis, mitophagy, and redox homeostasis, including PGC-1α, sirtuins, Nrf2 signaling, and mitophagy-related machinery [62,63,91].

EVs may improve mitochondrial membrane potential, lower ROS accumulation, and promote ATP synthesis in aged reproductive cells by delivering these chemicals in a specific manner. Conversely, age-related alterations in EV cargo, characterized by increased inflammatory mediators and reduced antioxidant capacity, may exacerbate mitochondrial dysfunction and adversely affect reproductive outcomes [63].

### 8.4. EVs as Modulators of Cellular Senescence

Additionally, EVs control the spread of senescence-associated signals and cellular senescence. Pro-inflammatory and pro-oxidant substances found in EVs generated by senescent cells have the potential to increase SASP activity and trigger secondary senescence in nearby cells. In contrast, EVs derived from young, metabolically healthy, or stem-cell-rich sources have demonstrated the capacity to suppress senescence markers, reduce OS, and restore mitochondrial function in experimental settings [62,71].

These opposing EV populations suggest that the balance between pro-senescent and anti-senescent vesicle signaling may critically influence reproductive lifespan.

### 8.5. A Conceptual Model of the Gut–EV–Mitochondria Axis

Based on current evidence, we propose an emerging gut–EV–mitochondria axis as a unifying framework linking microbial metabolism, EV-mediated communication, mitochondrial quality control, and reproductive aging [22]. In this model, gut-derived metabolites modulate systemic redox balance and influence EV biogenesis and cargo composition. These EVs, in turn, deliver antioxidant enzymes, mitochondrial regulators, and regulatory RNAs to reproductive tissues, where they support mitochondrial integrity, limit OS, and suppress senescence pathways [116,117].

**Figure 1:**
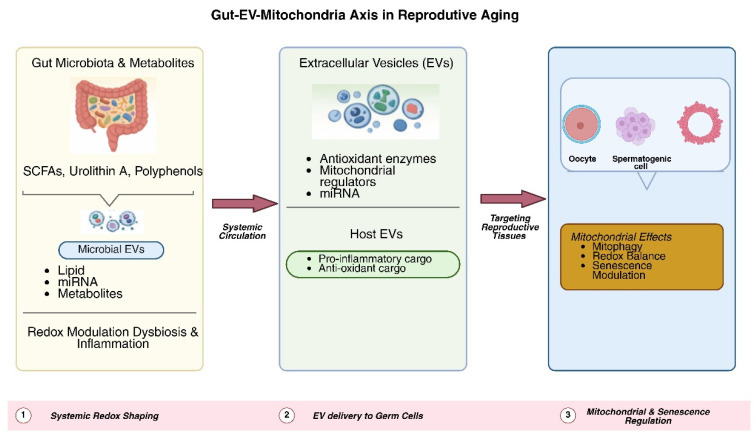
Schematic overview illustrating how gut microbiota–derived metabolites and extracellular vesicles influence mitochondrial function, oxidative stress, and senescence pathways in reproductive tissues, thereby contributing to reproductive aging.

EV signaling may shift toward pro inflammatory and pro senescent profiles in response to disruptions of this axis, such as those induced by dysbiosis, metabolic stress, or aging, thereby accelerating mitochondrial dysfunction and reproductive decline [118,119]. This integrated framework, summarized in Table 4, integrates metabolic, vesicle-mediated, and mitochondrial mechanisms into a cohesive model of reproductive aging. Notably, while microbiota-derived EVs clearly modulate immune and redox pathways in multiple organ systems, direct studies examining their trafficking to reproductive tissues and their effects on germ cell mitochondria remain limited, underscoring an important area for future research. This proposed stepwise model of gut microbiota–derived metabolites and EV-mediated regulation of mitochondrial function and senescence in reproductive tissues is summarized in Figure 1.

## 9. Therapeutic Implications and Future Directions

Accordingly, most therapeutic concepts discussed below are supported primarily by preclinical or early translational evidence, with clinical application in reproductive medicine still under development. New opportunities for therapeutic intervention in reproductive aging have been made possible by advances in our understanding of the relationships between metabolites generated from the gut microbiota, EV signaling, mitochondrial function, and OS. Targeting several elements of the gut–EV–mitochondria axis may provide chances to maintain reproductive function and prolong reproductive healthspan, even if many of these treatments are still in the experimental or early translational stages [123,124].

### 9.1. Microbiome Modulation to Support Mitochondrial Redox Balance

Accessible methods to affect OS and mitochondrial function in reproductive tissues include dietary and microbiome-targeted therapies. Diets rich in dietary fiber, polyphenols, and fermented foods promote the production of microbiome-derived metabolites, including urolithins and short-chain fatty acids. These effects may be further enhanced by probiotics and prebiotics that promote the abundance of bacterial populations capable of producing butyrate or urolithin [125,126].

By delivering specific antioxidant or mitochondrial modulating compounds without the use of live bacteria, postbiotics, defined as bioactive microbial metabolites or components, represent a more controlled therapeutic approach. While emerging evidence supports beneficial effects on systemic redox balance and inflammatory regulation, direct evidence for improved reproductive outcomes remains limited, and well-designed clinical studies are required to determine their effectiveness in improving oocyte quality, sperm function, and reproductive outcome [127,128].

### 9.2. EV-Based Therapeutic Approaches

Because EVs can carry bioactive compounds with low immunogenicity, they are increasingly being investigated as therapeutic tools in regenerative medicine. EVs made from mesenchymal stem cells or reproductive tissues have shown promise in reducing OS, restoring mitochondrial function, and suppressing senescence markers in testicular and ovarian cells in reproductive models [59,129].

A potential but experimental strategy is the use of engineered EVs that are intended to transport particular antioxidant enzymes, mitochondrial regulators, or regulatory microRNAs [130]. In assisted reproduction situations, these tactics might be especially useful for promoting embryo viability, maintaining sperm mitochondrial integrity, or improving oocyte maturation. However, before EV-based treatments can be implemented in clinical settings, challenges related to targeting specificity, dosage, safety, and large-scale production must be addressed [131,132].

### 9.3. Targeting Senescence and Mitochondrial Quality Control

Senotherapeutic strategies aimed at reducing the burden of senescent cells or modulating the senescence-associated secretory phenotype are gaining attention due to the central role of cellular senescence in reproductive aging. Quercetin, fisetin, and resveratrol are natural compounds with senomorphic and antioxidant properties that have shown beneficial effects in experimental models [133,134].

Another intriguing strategy is the activation of mitochondrial quality-control mechanisms, specifically mitophagy. Clinical studies outside reproductive medicine have demonstrated the safety and efficacy of urolithin A, a microbiome-derived metabolite that promotes mitophagy [39]. Whether similar benefits extend to germ cells and reproductive tissues remains to be determined, and further investigation is warranted to evaluate their potential role in reproductive aging. Redox-sensitive pathways, such as Nrf2, sirtuins, and AMPK signaling, may also be pharmacologically modulated to enhance mitochondrial resilience and reduce oxidative damage in reproductive organs [135]. Importantly, EV-mediated delivery of antioxidant enzymes, mitochondrial regulators, and senescence-modulating microRNAs represents a plausible mechanistic route through which systemic metabolic or senotherapeutic interventions could influence germ cell mitochondrial integrity and reproductive aging.

### 9.4. Applications in ART

Opportunities to improve assisted reproductive technologies (ARTs) are also provided by the gut–EV–mitochondria system. ART results may be improved by altering the mother’s microbiota before ovarian stimulation, adding antioxidant metabolites or EVs to in vitro culture systems, and using EV-based biomarkers to evaluate gamete or embryo quality [124].

For instance, EVs loaded with antioxidant cargo may be able to lessen OS during extended embryo culture or in vitro oocyte maturation. Similarly, interventions that enhance mitochondrial activity may support improved developmental competence of embryos from older individuals. While these approaches remain largely experimental, they illustrate the translational potential of incorporating vesicle-based and metabolic strategies into reproductive medicine [136].

### 9.5. Future Research Directions

Despite substantial advances, several important challenges remain unresolved. First, more research is needed to understand the mechanisms controlling the transfer of EVs originating from the gut and microbiota to reproductive organs [137]. Second, it is unknown to what degree probiotic or nutritional therapies can consistently change the composition of EV cargo in humans. Third, defining the timing, tissue specificity, and reversibility of mitochondrial dysfunction and senescence in reproductive organs will be critical for optimizing therapeutic strategies [138].

Lastly, to assess the safety, effectiveness, and long-term effects of microbiome-targeted medicines, postbiotics, senotherapeutics, and EV-based interventions in reproductive aging, well planned clinical trials are crucial. Bridging these experimental findings with clinical practice will be necessary to translate the gut–EV–mitochondria paradigm into actionable strategies for preserving fertility and enhancing reproductive healthspan [128,139].

## 10. Conclusions

The gradual accumulation of OS, mitochondrial dysfunction, and cellular senescence in ovarian, testicular, and reproductive tract tissues drives reproductive aging. Although these systems have historically been examined separately, there is growing evidence that systemic metabolic and intercellular communication networks have an impact on them.

This narrative review proposes an emerging gut–EV–mitochondria axis as a unifying framework for understanding reproductive aging by integrating existing research on metabolites generated from the gut microbiota, EV-mediated signaling, and mitochondrial redox control. Together with EVs that carry antioxidant enzymes and mitochondrial regulators, microbiome-derived compounds with antioxidant and mitophagy-enhancing qualities may enhance mitochondrial integrity, reduce oxidative damage, and inhibit senescence pathways in reproductive cells.

Redox and vesicle-mediated signaling may shift toward pro-inflammatory and pro-senescent patterns in response to disruptions of this axis, such as dysbiosis, metabolic stress, or aging. These changes may accelerate mitochondrial decline and reduce fertility. In contrast, therapies that modulate EV-mediated antioxidant signaling or restore microbial metabolic balance may help preserve reproductive function and extend reproductive health.

To establish the translational significance of microbiome- and EV-based therapies, as well as to assess their safety and effectiveness in reproductive medicine, more experimental and clinical research is needed to clarify the processes driving gut–EV communication with reproductive organs. Further development of this paradigm may guide future research and therapeutic design. Such approaches could reduce OS, enhance mitochondrial resilience, and support fertility across the reproductive lifespan.

## Figures and Tables

**Table 1:** This table summarizes the major microbiome-derived metabolites, their mitochondrial mechanisms, and their known impacts on ovarian, testicular, and embryonic function.

Metabolite	Primary Microbial Source	Mitochondrial/Redox Mechanism	Effects on Reproductive Tissues
Urolithin A [12,39]	Ellagitannin-converting bacteria (e.g., *Gordonibacter*)	PINK1–Parkin mitophagy; mitochondrial turnover; ROS reduction	Oocyte mitochondrial integrity; sperm motility; delayed reproductive senescence
Butyrate (SCFA) [44,53]	Butyrate-producing Firmicutes	HDAC inhibition; ↑ PGC-1α/NRF1/TFAM; mitochondrial biogenesis; Nrf2–glutathione	Folliculogenesis support; granulosa cell redox balance; sperm mitochondrial protection
Indole derivatives [47,48]	Tryptophan-metabolizing bacteria	AhR activation; Nrf2 signaling; inflammatory suppression; mitochondrial stabilization	Oocyte competence; testicular cell protection; steroidogenic stability
Polyphenol metabolites [54,55]	Microbial metabolism of dietary polyphenols	Membrane stabilization; SIRT1/SIRT3 activation; bioenergetic efficiency; lipid peroxidation reduction	Oocyte maturation; sperm chromatin integrity; embryo developmental support

**Table 2:** This is an overview of EV origins in reproductive tissues, their antioxidant/mitochondrial cargos, and their contributions to gamete and embryo function.

EV Source	Characteristic Cargo	Redox/Mitochondrial Mechanism	Reproductive Function
Follicular fluid EVs [77,79]	miRNAs, SOD, catalase, growth factors	Reduce oocyte ROS; support mitochondrial potential; regulate meiotic stability	Enhance oocyte competence and follicular development
Oviductal EVs [80,81]	Lipids, metabolic enzymes, small RNAs	Support early embryo antioxidant capacity; stabilize mitochondrial activity	Promote fertilization and early embryo cleavage
Seminal plasma EVs [84,90]	Antioxidant enzymes, membrane proteins	Protect sperm mitochondria from oxidative damage; stabilize membrane potential	Enhance sperm motility and fertilization potential
Epididymosomes [85,86]	miR-lipid complexes, mitochondrial regulators	Transfer antioxidant cargo to maturing sperm	Improve sperm chromatin quality and motility
Endometrial EVs [83,91]	Cytokines, miRNAs	Regulate redox tone at implantation site	Support endometrial receptivity
Embryo-derived EVs [88,89]	mtDNA, metabolic RNAs	Reflect embryonic redox status; modulate maternal signaling	Influence implantation and maternal–embryo communication

**Table 3:** Key interactions between senescence pathways, mitochondrial dysfunction, and specific reproductive impairments during aging.

Tissue/Cell Type	Key Senescence Markers	Mitochondrial Defects	Reproductive Consequences
Granulosa cells [93,100]	↑ p16, p21; SASP cytokines	Reduced membrane potential; increased mtDNA damage	Impaired folliculogenesis; reduced oocyte competence
Oocytes [101,102,103]	Meiotic spindle instability; telomere shortening	Impaired mitophagy; defective ATP production	Poor embryo development; higher aneuploidy rates
Sertoli cells [104]	SASP secretion; DNA damage foci	Mitochondrial swelling; ROS leakage	Disrupted sperm maturation; impaired blood–testis barrier
Leydig cells [105,106]	p53/p21 activation	Reduced oxidative phosphorylation; ROS accumulation	Decreased testosterone synthesis
Spermatocytes/spermatozoa [107]	Chromatin fragmentation; apoptosis	Loss of membrane potential; ROS-mediated damage	Reduced motility and fertilizing ability

**Table 4:** This table integrates current evidence describing how gut-derived metabolites, host- and microbiota-derived EVs, mitochondrial regulatory pathways, and senescence mechanisms interact to influence reproductive aging.

Component	Key Elements	Mechanisms Involved	Impact on Reproductive Tissues
Gut-derived metabolites [39,120]	Urolithin A, SCFAs, tryptophan metabolites, polyphenol derivatives	Stimulate mitophagy and mitochondrial biogenesis; reduce ROS; activate Nrf2, SIRT1, and PGC-1α pathways; modulate EV cargo loading	Improve oocyte mitochondrial quality; reduce granulosa cell OS; enhance sperm mitochondrial membrane potential; delay senescence in reproductive tissues
Host-derived EVs [79,121]	EVs from intestinal epithelium, immune cells, circulation	Deliver antioxidant enzymes (SOD, catalase, GPX); transport microRNAs targeting mitochondrial pathways; suppress SASP	Protect gametes from oxidative injury; stabilize mitochondrial membranes; support early embryo metabolic competence
Microbiota-derived EVs [19,61]	Bacterial vesicles carrying metabolites, lipids, RNAs	Enter systemic circulation; regulate immune and redox pathways; influence mitochondrial signaling	Modify ovarian and testicular redox tone; potentially affect folliculogenesis and sperm maturation
Mitochondrial pathways [39]	Mitophagy, biogenesis, mtDNA repair, oxidative phosphorylation	Remove damaged mitochondria; improve membrane potential; reduce leakage-derived ROS	Enhance gamete viability; support proper meiotic spindle formation; maintain sperm chromatin integrity; increase embryo developmental potential
Senescence pathways [61,122]	SASP factors, p16/p21 signaling, DNA damage responses	EVs suppress SASP; reduce mitochondrial ROS–induced damage; improve stress-response signaling	Lower accumulation of senescent granulosa, stromal, and Sertoli cells; maintain reproductive tissue function with age
Dysbiosis-associated disruption [19,120]	Reduced SCFA production; diminished urolithin synthesis; altered EV cargo	Increased pro-inflammatory vesicles; impaired mitophagy; elevated ROS; mitochondrial destabilization	Accelerated ovarian reserve decline; reduced sperm quality; impaired embryo development and implantation potential

## Data Availability

No new data were created or analyzed in this study. Data sharing is not applicable to this article.
